# Acceptability of orodispersible films for delivery of medicines to infants and preschool children

**DOI:** 10.1080/10717544.2017.1370512

**Published:** 2017-08-31

**Authors:** Mine Orlu, Sejal R. Ranmal, Yucheng Sheng, Catherine Tuleu, Paul Seddon

**Affiliations:** a Department of Pharmaceutics, School of Pharmacy, University College London, London, UK;; b Royal Alexandra Children's Hospital, Sussex, UK

**Keywords:** Paediatric drug delivery, infant, medicine acceptability, questionnaire, orodispersible film

## Abstract

Orodispersible films (ODFs) possess potential to facilitate oral drug delivery to children; however, documentation of their acceptability in this age group is lacking. This study is the first to explore the initial perceptions, acceptability and ease of use of ODFs for infants and preschool children, and their caregivers through observed administration of the type of dosage form. Placebo ODFs were administered to children stratified into aged 6 to 12 months, 1 year, 2 years, 3 years, 4 years and 5 years old and into those with an acute illness or long-term stable condition in hospital setting. Acceptability of the dosage form and end-user views were assessed by (a) direct observation of administration, (b) questionnaires to caregivers and nurses, and (c) age-adapted questionnaires for children aged 3 years and over. The majority of children (78%) aged 3 years and over gave the ODF a positive rating both on verbal and non-verbal scales. Despite little prior experience, 78% of caregivers expressed positive opinion about ODFs before administration. After the ODFs were taken, 79% of infant caregivers and 86% caregivers of preschool children positively rated their child’s acceptance of the ODF. The intraclass correlation coefficient value was 0.92 showing good agreement between ratings of caregivers and nurses. ODFs showed a high degree of acceptability among young children and their caregivers. If drug loading permits, pharmaceutical companies should consider developing pediatric medicines in this format. The methodology described here is useful in assessing the acceptability of active ODF preparations and other dosage forms to children.

## Introduction

The European Union Paediatric Regulation by the European Medicines Agency (EMA) is about to celebrate its 10th anniversary. Its aim is to incentivise companies to generate evidence towards regulatory approval of medicines for managing childhood conditions. One critical parallel objective is the development of age-appropriate dosage forms to ensure that children will have access to dosage forms with a positive benefit-risk balance. However, there is still a paucity of data on end-user requirements and acceptability of type of dosage form, which limits choice in the development of pediatric medicines.

Orodispersible films (ODFs) are postage stamp sized strips of thin polymeric films formulated to quickly disintegrate in the mouth when placed onto the tongue (Hoffmann et al., 2011). ODFs have been presented as a promising type of dosage form for nonstandard patient populations who experience difficulties swallowing oral medicines, such as children and older adults (Slavkova & Breitkreutz, 2015). An increasing number of studies have focused on their pharmaceutical development, including formulation optimization, characterization, assessment of mechanical properties, and efficient taste masking of the drug to be loaded into the film (Hoffmann et al., 2011; Preis et al., 2013; Borges et al., 2015; Brniak et al., 2015; Visser et al., 2015a). ODFs have been recently proposed as customized small scale pharmacy preparations for individualized pharmacotherapy (Visser et al., 2015b). ODFs are an attractive solid dosage form option for administering medicines to young children given the problems with conventional tablets and capsules, including difficulty in swallowing. An Ondansetron ODF has been marketed in the UK since 2010 for the management of chemotherapy-induced and post-operative nausea and vomiting in children (Medicines.org.uk, 2017). However, there are currently no published empirical studies demonstrating the acceptability of ODFs among children and their caregivers in acute or non-acute situations.

Appropriate and acceptable dosage forms are of vital importance in pediatric prescribing. Patient acceptability has been defined as ‘the overall ability and willingness’ of the patient and their caregiver to administer the medicines as intended (EMA, 2013). This can have a major effect on adherence and, therefore, effectiveness of treatments. The EMA requires evidence of the suitability of dosage forms to be included in all Paediatric Investigation Plans (PIPs) where possible, and for patient acceptability to be assessed during pharmaceutical and clinical development. Nevertheless, methodologies for assessing acceptability are still fragmented, and more research is needed to contribute towards the development of a globally harmonized approach (Kozarewicz, 2014; van Riet-Nales et al., 2016).

The primary aim of the STAMP (**S**tudy into **T**hin orodispersible film **A**cceptability as **M**edicine for **P**reschool children) study was to explore the acceptability of ODFs by infants (aged 6 months to 23 months) and preschool children (2 to 5 years). The secondary objective was to develop a methodology for testing acceptability of ODFs in young children, and to explore the agreement between individuals rating acceptability after observing children’s behavior and reactions to orally administered dosage forms. The study involved combining questionnaire responses with observed administration of the placebo dosage form, to obtain a validated measure of end-user acceptability.

## Methods

STAMP was a single site, open label trial using placebo ODFs (commercially manufactured by Bouty SpA, Milano, Italy). The ODFs were 6cm^2^ in size (3cmx2cm), flexible, opaque white, odorless and slightly sweetened, with average weight of 85.0 mg/film ±5.0%. The study participants were children aged 6 months to 5 years 11 months, together with their main caregivers in pediatric outpatient and emergency department settings at the Royal Alexandra Children's Hospital, Brighton, UK.

Participants were stratified into 6 age groups, including *n* = 8 aged 6 to 12 months, *n* = 25 aged 1 year old, *n* = 33 aged 2 years old, *n* = 20 aged 3 years old, *n* = 11 aged 4 years old and *n* = 13 aged 5 years old.

Approximately half the participants were recruited from the outpatient department and half from the emergency department, representing both stable and acutely ill children, respectively. Ethical approval was granted by the NHS National Research Ethics Service (NRES) (REC 13/LO/0134).

**Figure 1.**
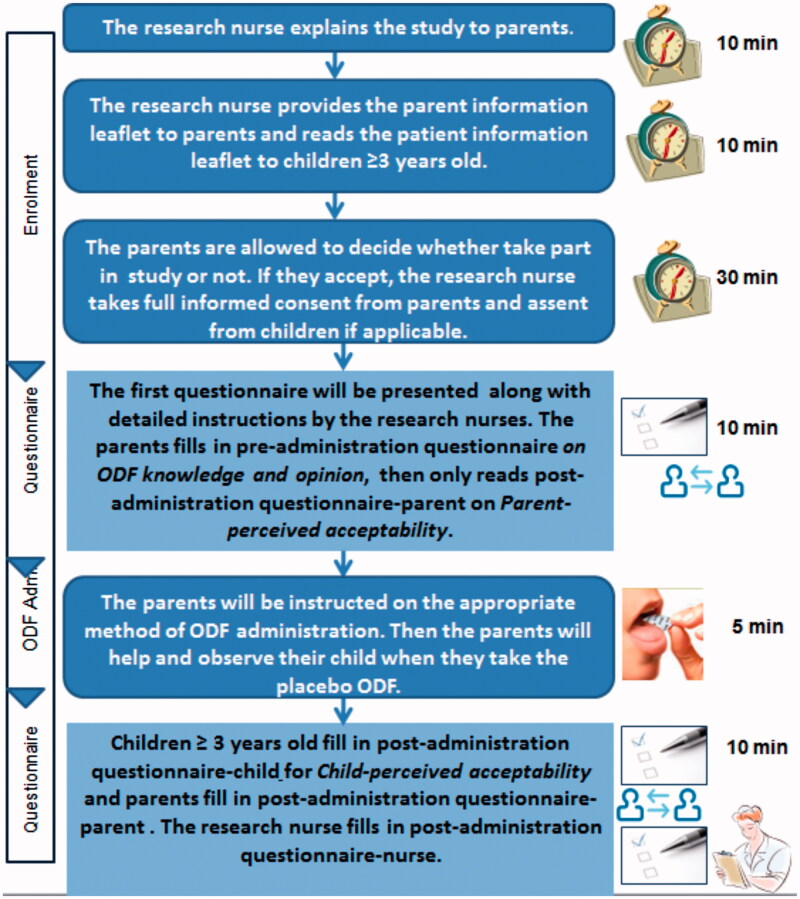
Flowchart for STAMP study.

The study design is illustrated in Figure 1. Pre- and post-administration questionnaires were developed to capture end-user perceptions of acceptability as described below:

**Figure 2.**
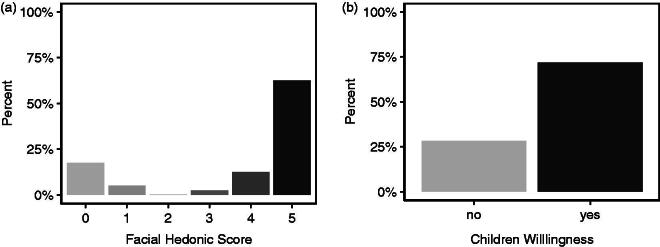
Percentage of responses from children aged 3 years and older to (a). ‘How much did you like the medicine wafer?’ ranging from 0: ‘not at all’ to 5: ‘very much’. (b). Would you be happy to take it again?

**Figure 3.**
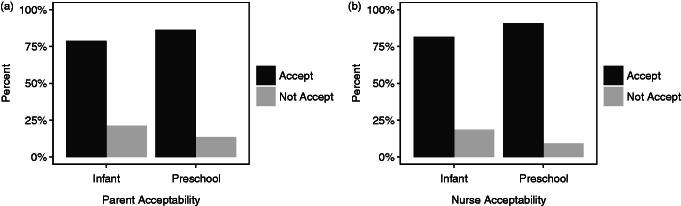
Percentage of responses from a. caregivers and b. nurses reporting ODFs acceptable (≥5 on the Medication Acceptance Scale) and not acceptable (<5 on the Medication Acceptance Scale) for infants and preschool children.

**Figure 4.**
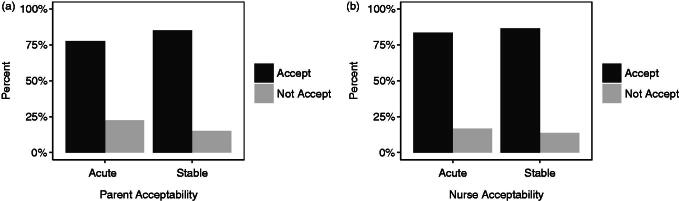
Percentage of responses from (a) caregivers and (b) nurses reporting ODFs acceptable (≥5 on the Medication Acceptance Scale) and not acceptable (<5 on the Medication Acceptance Scale) for infants and children in acute and stable (chronic) settings.

1. Pre-administration caregiver questionnaire: completed by the caregiver prior to administration of the ODF. This captured participant demographics including the child’s age, gender, and feeding status (e.g. puree, soft chew food, solid food), including presence of any existing feeding problem. It also sought the caregivers’ knowledge, experience and initial perceptions of ODFs as a means of delivering medicines.
2. Post-administration children’s questionnaire: completed by children aged 3 years and older following administration of one placebo ODF. This questionnaire used a five-point facial hedonic scale anchored with statements as to whether the child liked taking the ODF, ranging from ‘not at all’ to ‘very much’. A second question assessed the participants’ willingness to take an ODF again.
3. Post-administration caregiver questionnaire: completed independently by caregivers after observing administration of the ODF. This included 5-items from the Medication Acceptance Scale (MAS) recording observed child behaviors and reactions to ODF administration (Kraus et al., 1999). The scale measures five behavioral distress items including cry, facial expression, body movement/level of agitation, reaction to placement in the mouth, and swallowing of the medication. This questionnaire also captured any concerns, and the caregivers’ willingness to administer ODFs again in the future.
4. Post-administration nurses questionnaire: completed independently by a research nurse after observing administration of the ODF. This questionnaire also incorporated the MAS.

Acceptability of the ODF was measured based on (1) successful administration of the ODF, (2) responses to the five-point facial hedonic scale by children aged 3 years and older, and (3) the total score on the 5-item MAS given by caregivers and nurses. For children aged 3 years and older, a score of 3 and above was regarded as `acceptable`. For the MAS scale, the same scoring system as Kraus et al. (1999) was used: 0 to 2 points were allocated for each item resulting in a total score between 0 and 10 (with 0 indicating the lowest level of acceptance and 10 the highest). The original description of MAS did not specify a threshold value for the definition of medicines acceptability (Kraus et al., 1999). In this study, a total score of 5 or above was regarded as acceptable. It was pre-specified that if a child scored 0 for the individual item on swallowing (indicating spitting out the entire dose or vomiting), the total MAS score of 0 was recorded without scoring the other 4 items of the questionnaire.

All data were collected on paper questionnaires at the clinical site and later transcribed electronically using Qualtrics (Provo, UT) for data analysis by the researchers.

### Sample size

The main aim of the study was to obtain descriptive data on the acceptability of ODF. There are few published studies in this area, and no comparison on acceptability with an existing dosage form is being made. A power calculation would not be suitable way of determining the number of participants for this study. The number of participants has, therefore, been determined from experience in previous similar studies, both from our group and from the literature. In a previous study by this group, this sample size provided adequate data to accurately characterize the acceptability of minitablets in a similar population (Thompson et al., 2009)

### Statistical analysis

Statistical analysis was performed using R version 3.0.2 (R Core Team, 2013). One-way intraclass correlation (ICC) with 95% confidence interval was performed to evaluate the extent of agreement of the post-administration MAS score between caregivers and nurses (McGraw & Wong, 1996). An ICC of 0.75 or above indicates good and above 0.9 excellent agreement (Sayinsu et al., 2007).

## Results

In total, 110 children were recruited in the study (59% male), of which 66 were infants and 44 were preschool children. The majority of the participants (91%) were reported to be eating normal family food while 6% had reached soft chew solids and the remaining 3% were reported to be eating purees. Fifty-seven children (52%) were recruited from the pediatric outpatients, representing those with a long-term stable condition, and 53 from the emergency department, representing those with an acute illness.

### ODF administration outcomes

The results of the swallowing of medicine section of MAS were used to assess children’s ability to take the ODF. Sixty-five percent of the caregivers and 62% of the nurses reported that children swallowed the ODF without loss. In 15% of the children, a partial loss of administered ODF was observed by caregivers and nurses: in these children the ODF became stuck to teeth, lips or palate resulting in it being partially spat out or removed by the child using their finger. No caregivers or nurses scored 0 for the individual swallowing item corresponding to the observation of spitting out entire dose of ODF or vomiting. One incident of gagging and one of vomiting was reported by caregivers but in both these instances, the research nurse deemed this to be due an underlying illness and not the dosage form itself.

### Acceptability of ODFs to children

Overall, 40 children aged 3 years and older self-reported acceptability using the post-administration questionnaire. The majority of children (78%) rated the dosage form ≥3 on the 5-point hedonic scale, and 63% reported that they ‘very much’ liked the ODF. Seventy-two percent reported a willingness to take an ODF again (Figure 2).

### Acceptability of ODFs as recorded by caregivers and nurses

Of the caregivers participating in the study, 66 had an infant child aged less than 2 years, and 79% of these respondents scored ≥5 on the MAS scale. The remaining 44 caregivers had a child at preschool age (≥2 years), and 86% scored ≥5 on the MAS scale. Over half of the caregivers of preschool children (52%) scored 10 on the scale indicating the highest level of acceptance. Nurses allocated a score ≥5 on the MAS scale in 83% of infants, and 91% of preschool children (Figure 3). The caregivers and research nurses showed excellent agreement on MAS scores with the ICC value 0.92 (95% CI: 0.886–0.945).

Acceptability of the dosage form was found to be similar between infants recruited from the emergency department and outpatients’ clinic according to the MAS scores. Among caregivers, 78% and 85% scored ≥5 on the total MAS for acute and stable conditions, respectively (Figure 4). Among nurses, this was closer at 83% and 86%, respectively.

### Perceptions of ODFs

Prior to commencing the study, 83% of caregivers were unfamiliar with ODFs. Among respondents with prior knowledge, familiarity of the dosage form most commonly in the form of breath fresheners and snoring treatments. Nevertheless, a large majority (78%) expressed an opinion that the dosage form would be a good way of giving medicines to their child and 84% were not concerned about the use of ODFs. 98% were willing to use ODF to administer medicines to their child. The most frequently perceived advantages of ODFs were ease of administration and reduced risk that the medicine would be spat out. Some caregivers reported an assumption that younger children would automatically start to chew items entering their mouth, but that children ≥3 years would be better able to understand the use of ODF without administering water. Overall, 82% of the caregivers reported that their child had taken a liquid medication in the past; 59% of these respondents felt that ODFs would be a better dosage form than administering medicine in liquid form, while 28% felt that they would be no different.

After administration of the placebo sample, most caregivers preserved their opinion about ODFs. Overall, 79% remained not concerned and 90% remained willing to administer ODF to their child. A larger majority (81%) reported that the dosage form was a good way of giving medicines to their child. The majority of caregivers (84%) felt that the ODF size (6cm^2^) was manageable, and that the appearance was suitable for their child. Negative comments mentioned color and flavor as features to be modified; with strawberry the most commonly suggested flavor. Caregivers also made suggestions regarding the shape of ODF such as child-friendly appealing shapes. Interestingly, some caregivers also suggested elongated shapes for the ease of administration.

## Discussion

This study showed a high degree of acceptability of ODFs among young children and their caregivers, whether assessed by caregiver questionnaire, caregiver or nurse observation, or by direct questionnaire to the older children. A previous study reported a high level of preference and adherence to filmstrips amongst healthy infants; however, this relied to be the solely self-reported behavior by the parents (Rodd et al., 2011). The STAMP study is the first to assess the acceptability of ODFs using self-reported responses from preschool children, and using an objective measure in the form of observed administration of the dosage form by a research nurse.

Amongst formulation scientists, there is awareness of inherent advantages of ODFs for special patient populations ranging from pediatric to bedridden and non-cooperative patients (Borges et al., 2015; Slavkova & Breitkreutz, 2015). This is light of the well-acknowledged problems of oral liquid dosage forms, such as medication errors (e.g. risk of under or over dosing due to inappropriate dosing and risk of poor therapeutic outcome due to difficulty in using oral administration device) as well as the lower acceptability of parenteral and rectal drug delivery. A recent review article raised the possibility of using ODFs from birth (van Riet-Nales et al., 2016). However, ODFs have been so far only used for niche clinical conditions. It is unclear why ODFs are not more widely used. One limitation is that a therapeutic effect must be achievable at the low doses that can be loaded into the polymeric film. The lack of established quality control/quality assurance criteria and related methodologies may be a further barrier. However, perhaps the biggest obstacle has been that the acceptability of ODFs to young children and their caregivers has not been explored in depth. The preference and perception of patients towards novel dosage forms is critical for the acceptability of the prescribed medicine. This translational study supports the potential of ODFs for broader pediatric indications and provides evidence to stimulate the interest of pharmaceutical industry to develop these.

The perception of the patient and caregiver about the dosage form itself plays a crucial role in the optimization of acceptability and the adherence to treatment (Matza et al., 2013). Previously our research group took the initiative in exploring the acceptability and suitability of placebo minitablets for preschool-aged children (Thompson et al., 2009). The positive outcome showing the ability of majority of youngest children to swallow minitablet has paid the way for the further academic and industrial trials with the aim of determining the acceptability of pediatric medicines (Mistry & Batchelor, 2017) hence positively influence the implementation of this new dosage form into manufacturing and clinical settings.

The questionnaires were adapted to the needs of the present study and improved with the involvement of lay members (National Institute of Health Research – Medicines for Children Research Network, Parent Representatives and Young Persons Advisory Group) at an early stage. This review enabled the format of questions to be appropriately designed for the targeted age group. In particular, involvement of young people improved the design of the artwork used in participant information leaflets which supported volunteer recruitment. It was interesting to note that particular care must be taken when phrasing questions to ensure they are correctly understood by child respondents. As an example, in the post-administration children’s questionnaire, respondents were asked ‘would you like to take it again?’ with the aim to determine if the respondent would be happy to receive the ODF again at some point in the future. However, the nurse reported that some younger children understood this to mean whether they would like to receive another ODF immediately and, therefore, responded negatively, although their response about the ODF administration itself was positive. This demonstrates the complexity in stating questions clearly to avoid any potential misinterpretation or unintentional response. Another study design limitation was the restricted diversity of the sample population recruited from one site. The caregivers completed the questionnaires in the presence of the nurse, and children (over 3) completed the questionnaire in the presence of their caregiver. It is possible that this may have biased the responses, and this issue could be explored in future studies. This is an exploratory study, with sample size based on previous similar studies. A larger study would be needed to confirm the study findings and to examine any differences in acceptability between the age strata. The data generated forms the basis for further development of this novel dosage form.

## Conclusions

This study demonstrates that ODFs are an acceptable, age-appropriate dosage form for infants and preschool children. The high degree of concordance between parental and nurse ratings suggest that this methodology is a reliable way to assess acceptability of novel dosage forms in this age group.
